# Supplementary material for: TNF is a potential therapeutic target to suppress prostatic inflammation and hyperplasia in autoimmune disease
Source: Nat Commun. 2022 Apr 19;13:2133. doi: 10.1038/s41467-022-29719-1 (PMC9018703; doi:10.1038/s41467-022-29719-1)
Supplement: Supplementary file 1 — Supplementary Information [file 41467_2022_29719_MOESM1_ESM.pdf]

Supplementary Information

"TNF is a Potential Therapeutic Target to Suppress Prostatic Inflammation and Hyperplasia in Autoimmune Disease"

This supplementary information contains 16 supplementary figures and 15 supplementary tables.

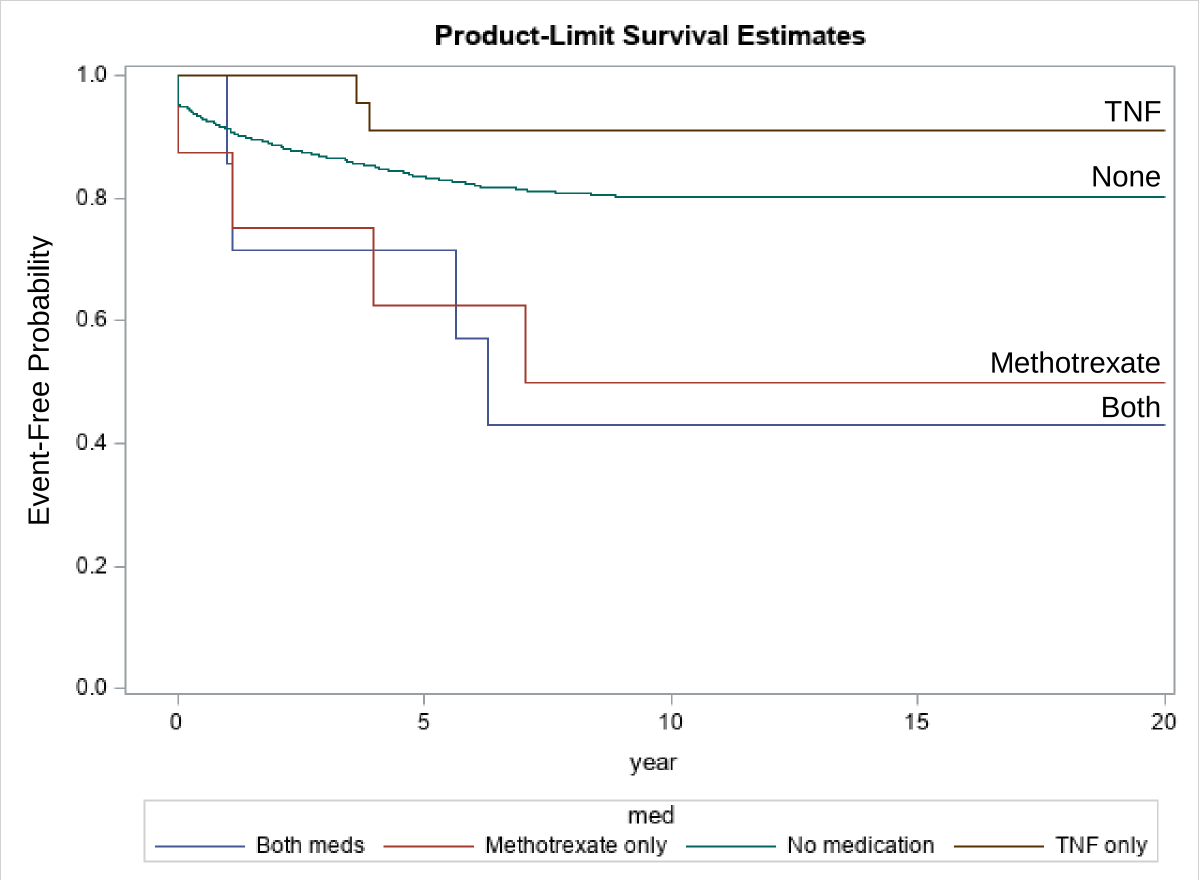

Supplementary Figure 1. Longitudinal analysis for patients free of BPH diagnosis based on medication use in men with a diagnosis of ulcerative colitis or Crohn's disease prior to BPH diagnosis (n=1242). The Kaplan-Meier event-free probability indicates the likelihood of remaining free of BPH diagnosis over time (log-rank test = 12.7; p=0.0054). Curves indicate medication use by patients, including both methotrexate and TNF-antagonists (blue), methotrexate only (red), no medication (green), and TNF-antagonists only (brown).

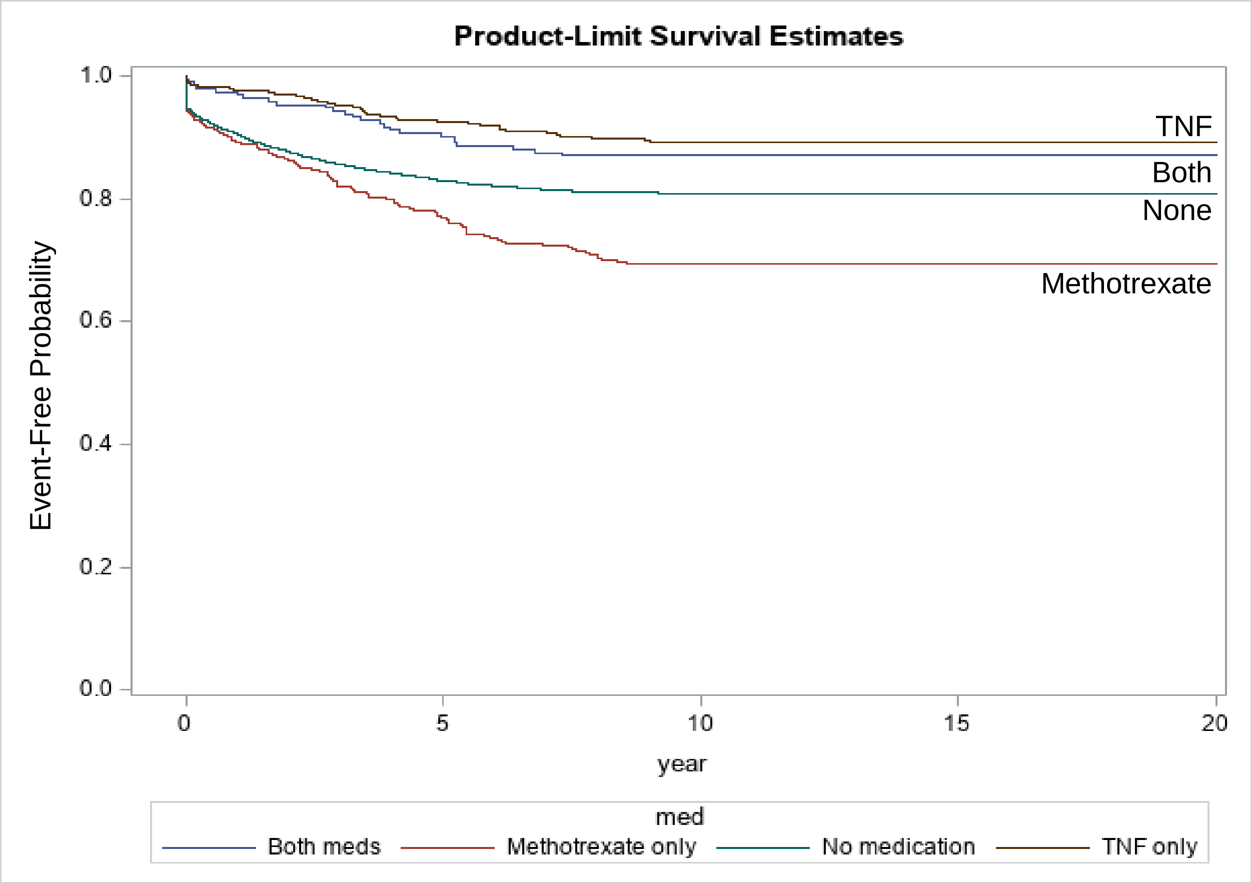

Supplementary Figure 2. Longitudinal analysis for patients free of BPH diagnosis based on medication use in men with an AI condition other than ulcerative colitis and Crohn's disease prior to BPH diagnosis (n=8023). The Kaplan-Meier event-free probability indicates the likelihood of remaining free of BPH diagnosis over time (log-rank test = 49.9; $p<0.0001$). Curves indicate medication use by patients, including both methotrexate and TNF-antagonists (blue), methotrexate only (red), no medication (green), and TNF-antagonists only (brown). Nine patients taking other treatments (T cell or interleukin-based therapies) were excluded from this analysis.

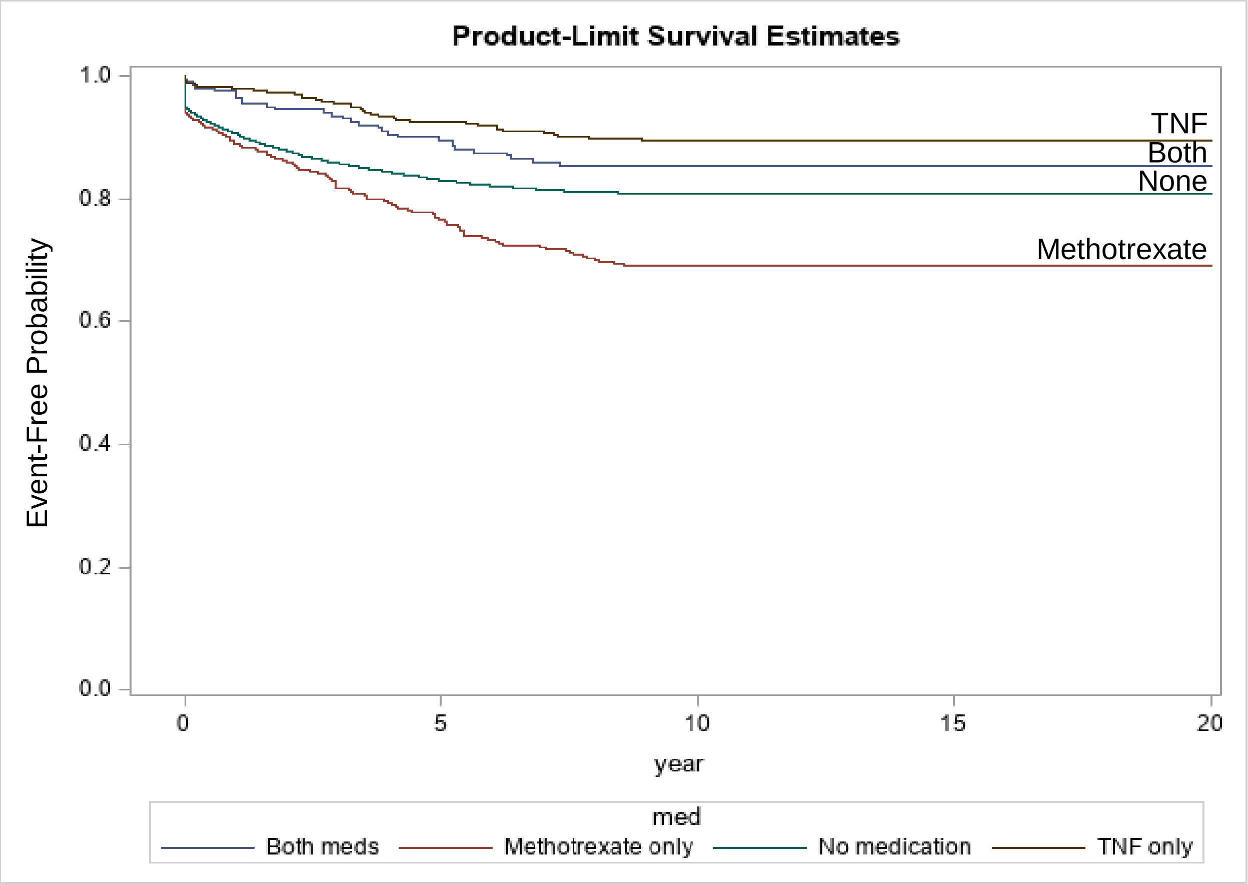

Supplementary Figure 3. Longitudinal analysis for patients free of BPH diagnosis based on medication use in patients with any AI condition prior to BPH diagnosis (n=9265). The Kaplan-Meier event-free probability indicates the likelihood of remaining free of BPH diagnosis over time (log-rank test = 52.4; $p<0.0001$). Curves indicate medication use by patients, including both methotrexate and TNF-antagonists (blue), methotrexate only (red), no medication (green), and TNF-antagonists only (brown). Nine patients taking other treatments (T cell or interleukin-based therapies) were excluded from this analysis.

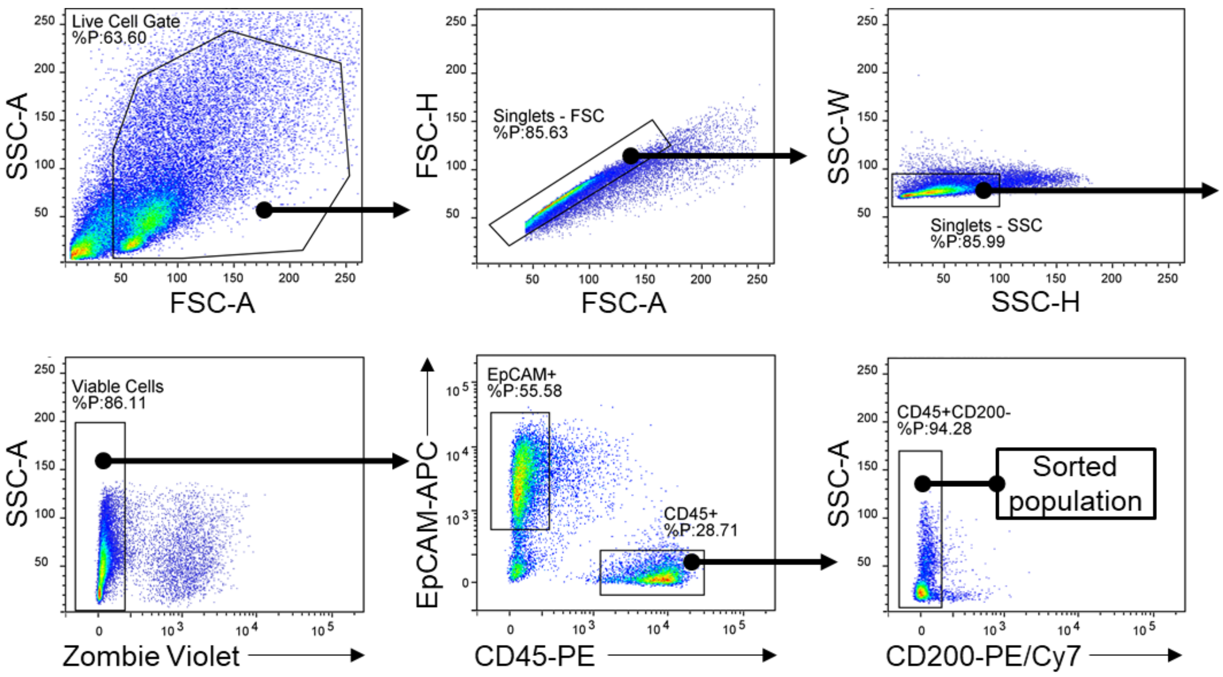

Supplementary Figure 4. Fluorescence activated cell sorting strategy for CD45$^+$ scRNA-seq analysis. Progressive gating strategy for isolation of CD45$^+$ cells following enzymatic digestion of human prostate tissues, in order from the top left panel to the bottom right panel. Zombie violet was used for exclusion of dead cells and EpCAM and CD200 were used to exclude epithelial and endothelial cells, respectively.

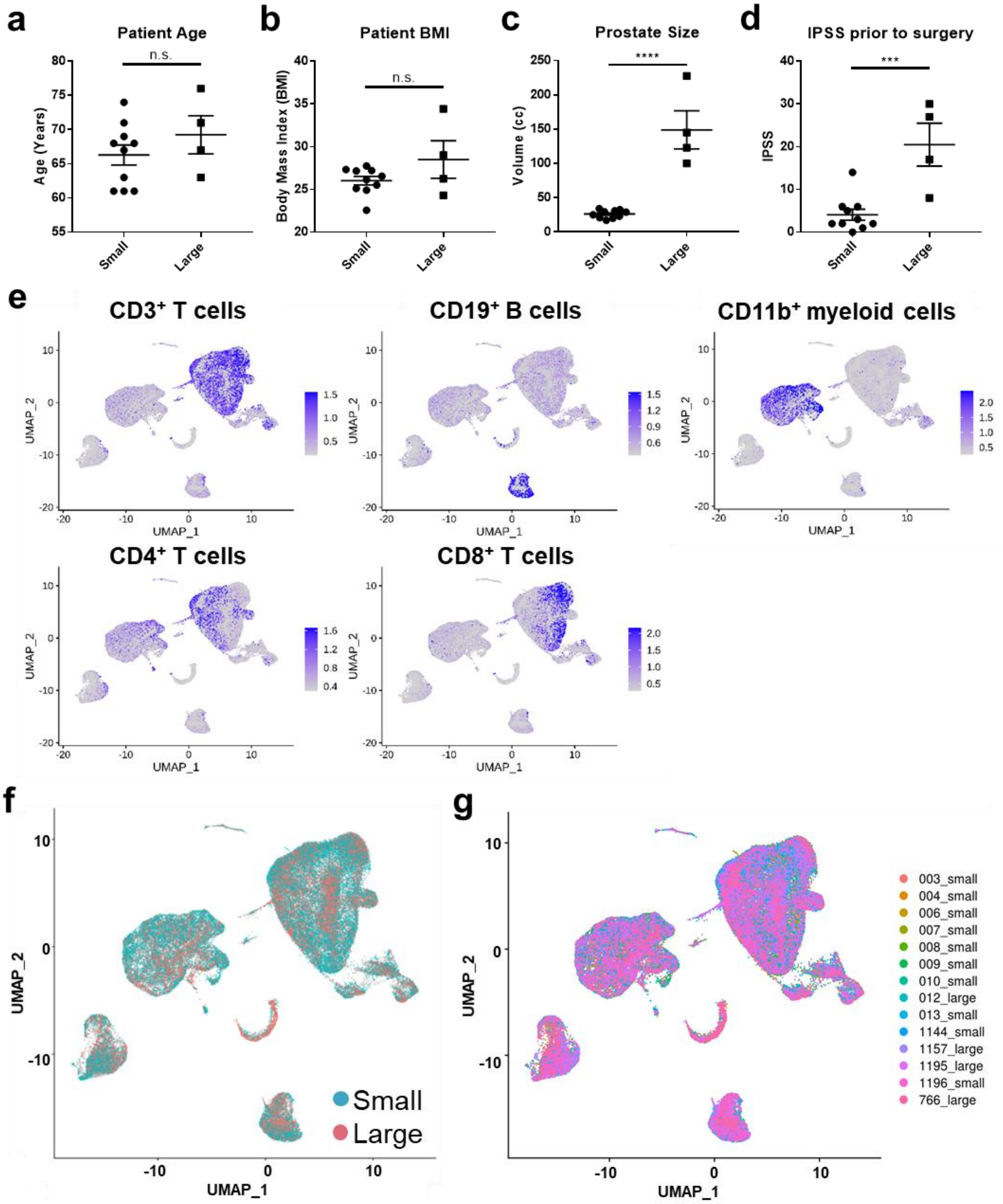

Supplementary Figure 5. Additional data relating to scRNA-seq data and patient characteristics. a) Patient age, **b)** patient BMI, **c)** prostate size estimation by TRUS or CT scan

(****$p<0.0001$), and **d)** IPSS prior to surgery (***$p=0.0007$) is shown for n=10 patients with small prostates and n=4 patients with large prostates. Statistical analysis was performed using a two-tailed t-test in (a-d). All patients are biologically independent. Error bars represent the mean +/- SEM for each graph. n.s.=not significant. Source data are provided as a Source Data file. **e)** Feature plots highlighting protein expression of CD3 ($CD3^+$ T cells), CD4 ($CD4^+$ T cells), CD8 ($CD8^+$ T cells), CD19 ($CD19^+$ B cells), and $CD11b^+$ (myeloid cells), where blue color indicates positive expression and gray color indicates no detectable expression of the indicated protein. CITE-seq analysis was conducted on three small and three large samples, although all 14 samples are displayed. **f)** UMAP plot of 69,850 BPH-associated cells, colored to highlight cells from small (blue) or large (pink) prostates, highlights the significant overlap between both populations. **g)** UMAP plot of 69,850 BPH-associated immune cells, colored to highlight cells from individual patients, indicates significant overlap among all individual patient samples.

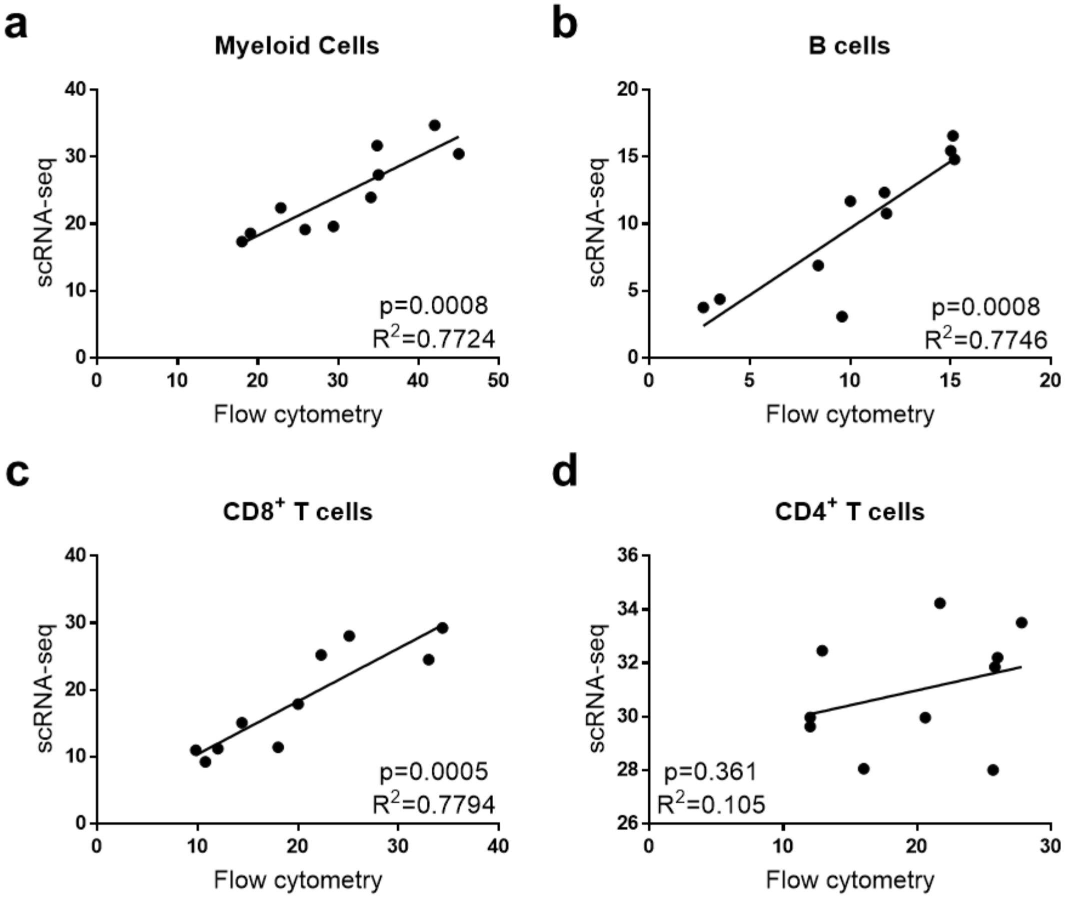

Supplementary Figure 6. Estimated immune cell type proportions by scRNA-seq analysis and flow cytometry are broadly concordant. Digested small (n=7) or large (n=3) prostate tissues were subjected to flow cytometry analysis after staining with antibodies against CD45, CD11b, CD19, CD4, and CD8. Sorted $CD45^+$ cells were subjected to scRNA-seq analysis from the same digested prostate tissues. Linear regression correlation analysis was conducted using Prism software and the percentages of $CD11b^+$ myeloid cells (a), $CD19^+$ B cells (b), $CD8^+$ T cells (c), and $CD4^+$ T cells (d), are shown as the estimated percentages of total $CD45^+$ cells via scRNA-seq versus flow cytometry. The respective p-values and R^2 values are indicated. Source data are provided as a Source Data file.

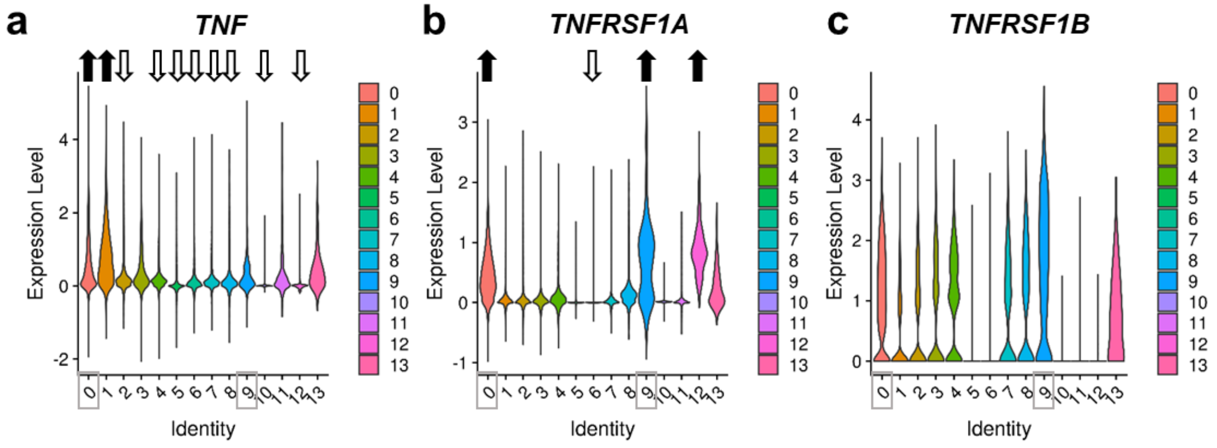

Supplementary Figure 7. Expression level of TNF, TNFRSF1A, and TNFRSF1B in BPH leukocytes. Violin plots indicate the normalized gene expression level (log[CPM]) for **a)** *TNF*, **b)** *TNFRSF1A*, and **c)** *TNFRSF1B* in each of the clusters identified in the $CD45^{+}$ scRNA-seq analysis. Each cluster is represented by a different color. Arrows indicate clusters with significantly up- or down-regulated gene expression (black or white arrows, respectively) compared to all other clusters, conducted using a two-tailed Wilcoxon rank sum test corrected for multiple testing using the Benjamini-Hochberg method. Gray boxes highlight clusters 0 and 9 as the macrophage subpopulations.

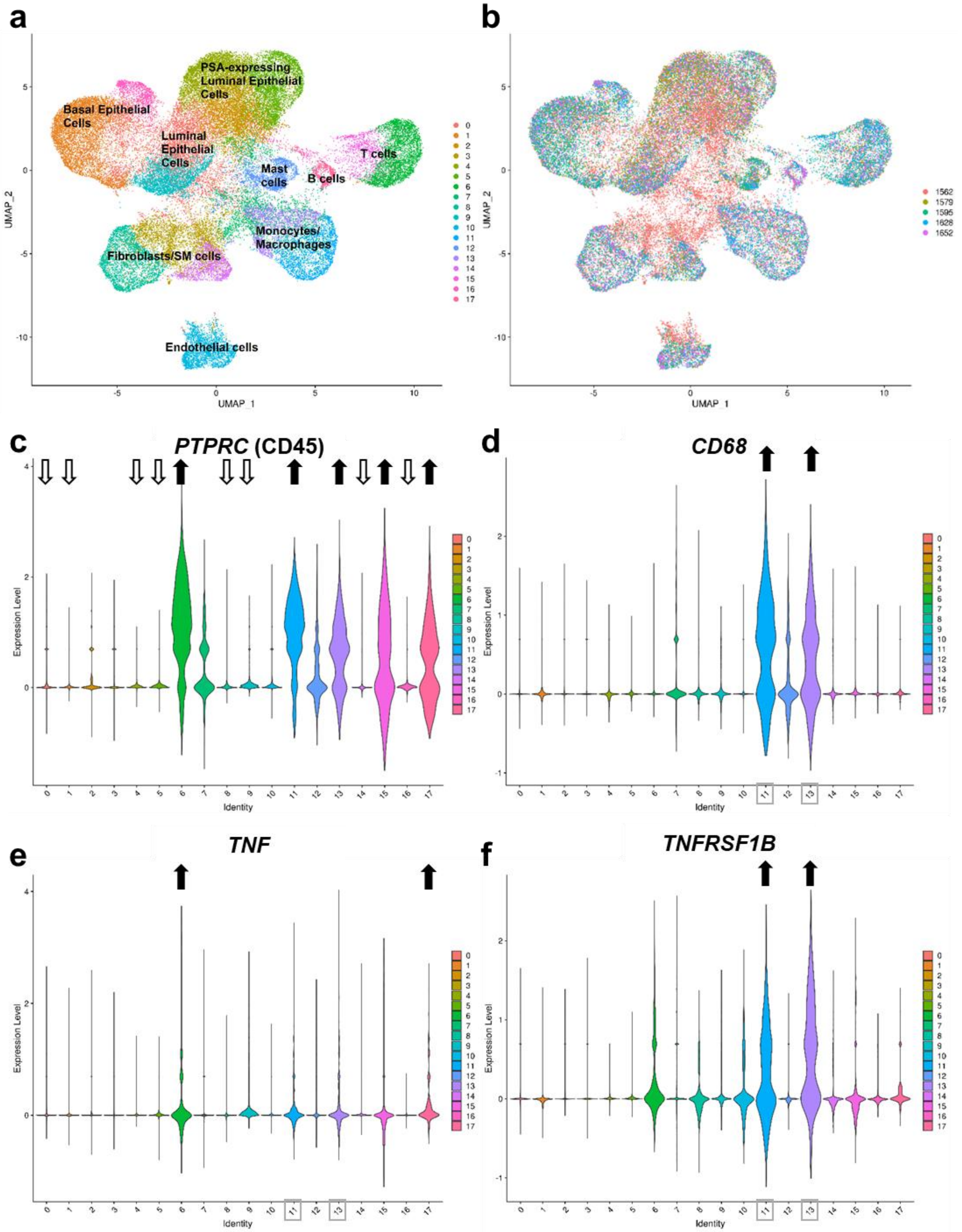

Supplementary Figure 8. Analysis of human BPH tissues via scRNA-seq highlights immune cells as both producers and responders to TNF. Prostate transition zone tissues from five BPH

patients were isolated after simple prostatectomy. Samples were digested, debris removed via centrifugation, and approximately 10,000 cells per sample loaded for scRNA-seq analysis using the 10X Chromium system. **a)** UMAP plot of 67,902 cells from a total of 5 patients indicates 18 unique clusters identified by differentially expressed genes. Colors indicate unique clusters and likely cell types label relevant clusters. **b)** UMAP plot of all BPH cells from (a), colored to highlight cells isolated from individual patients. **c-f)** Violin plots indicating expression of *PTPRC* (CD45) (c), *CD68* (d), *TNF* (e), and *TNFRSF1B* (TNFR2) (f) among each cell cluster. Colors identify unique cell clusters. Significantly altered expression of the indicated genes are indicated by either black (upregulated) or white (downregulated) arrows, determined using a two-tailed Wilcoxon rank sum test corrected for multiple testing using the Benjamini-Hochberg method. Of note, no reads were detected for *TNFRSF1A* gene expression in these cells.

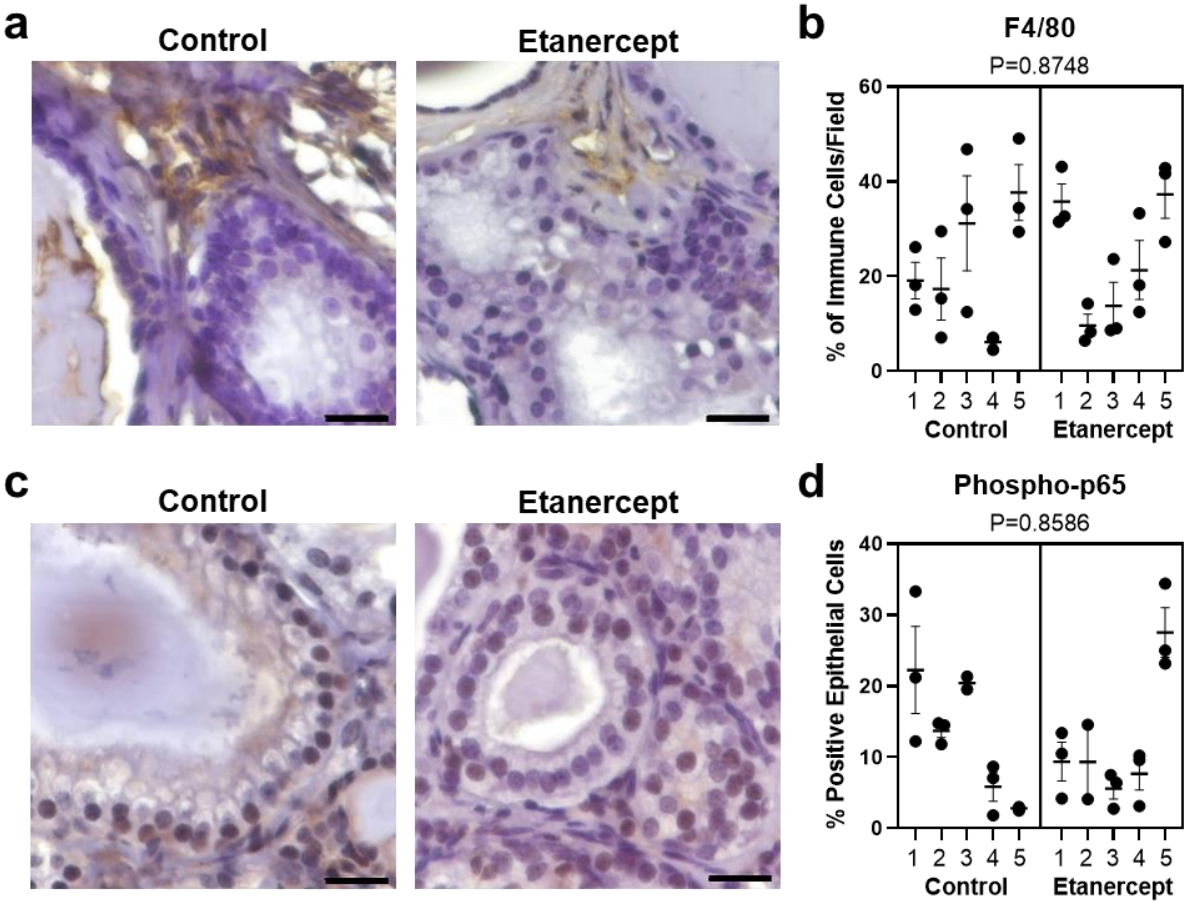

Supplementary Figure 9. TNF-antagonist treatment in Pb-PRL mice does not significantly alter macrophage infiltration or epithelial NFκB activity. a) Representative images of F4/80 staining in control or etanercept-treated mice, where brown color indicates positive staining. **b)** IHC staining counts for F4/80$^+$ cells, represented as the portion of all immune cells in the field. **c)** Representative images of phospho-p65 staining in control or etanercept-treated mice, where brown color in the nucleus indicates positive staining. **d)** Data presented indicates the percentage of phospho-p65 positive epithelial cells counted per field of view after IHC staining. **b, d)** Data indicate the mean +/- SEM of the percent positive cells in n=5 mice per group with counts from the indicated number of prostate tissue fields for each animal. Comparison of control and etanercept-treated groups for statistical purposes were conducted using nested t-test and the

reported p-values indicate the lack of significance of the treatment. Source data are provided as a Source Data file. Scale bars = 20µm.

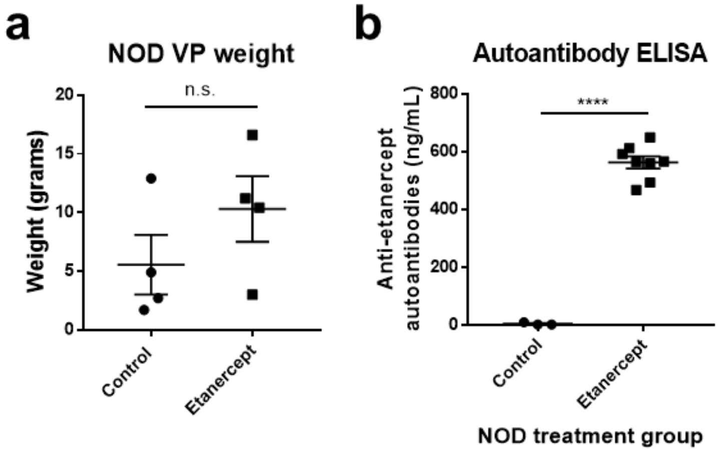

Supplementary Figure 10. NOD mice prostate weight and production of anti-etanercept autoantibodies. a) Dissected ventral prostate weights from control and etanercept-treated NOD mice (n=4 independent mice per group) indicate no significant change after treatment. **b)** ELISA on the serum of control (n=3 independent samples) and etanercept-treated (n=8 independent samples) NOD mice indicates the production of anti-etanercept autoantibodies in treated mice after the 5 week drug treatment. In both (a) and (b), analysis was completed using a two-tailed t-test and the error bars represent the mean +/- SEM. Source data are provided as a Source Data file. n.s. = not significant and **** indicates p<0.0001

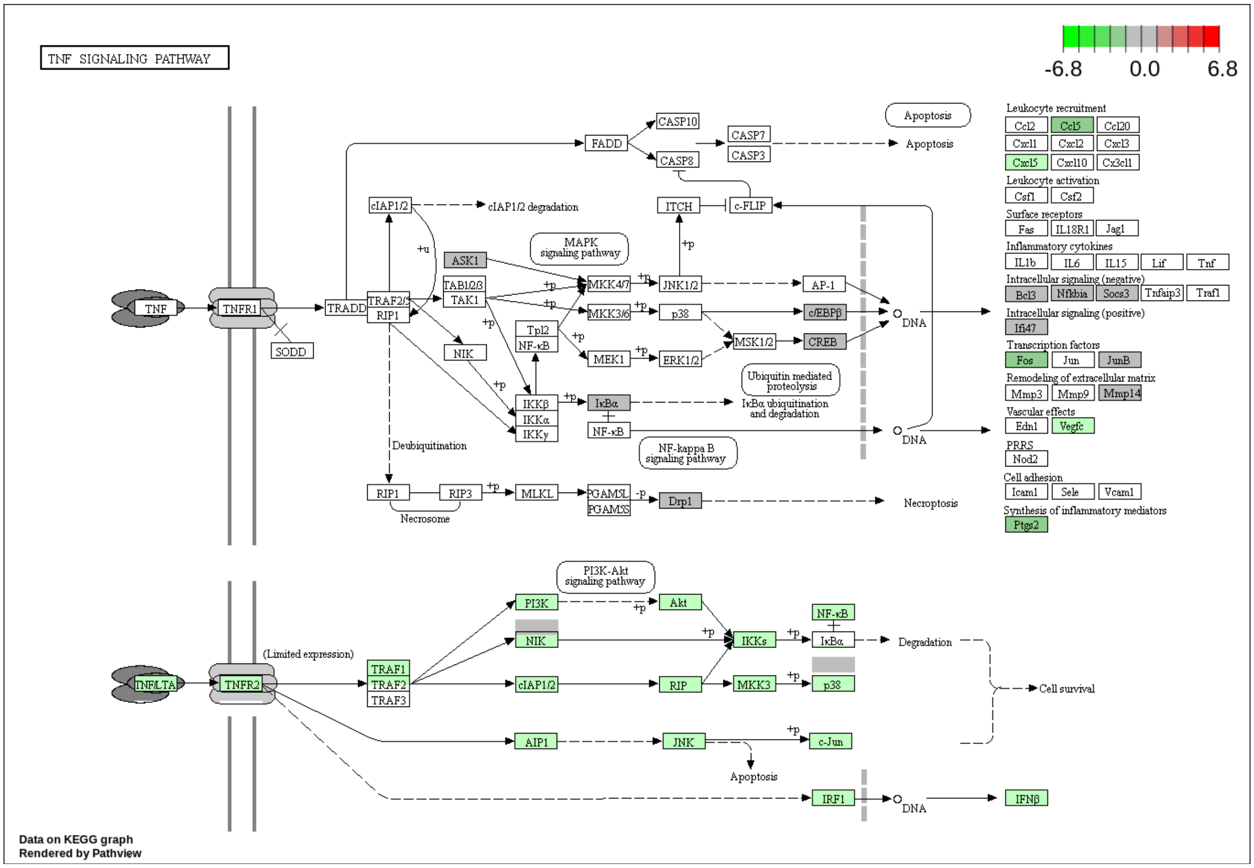

Supplementary Figure 11. TNF signaling pathway alterations in etanercept-treated versus control NOD prostate. KEGG enrichment analysis highlighted downregulation of a set of genes within the TNF signaling pathway. Downregulated genes are highlighted in green, where a greater intensity of color indicates greater downregulation. Units indicate standardized fold-change.

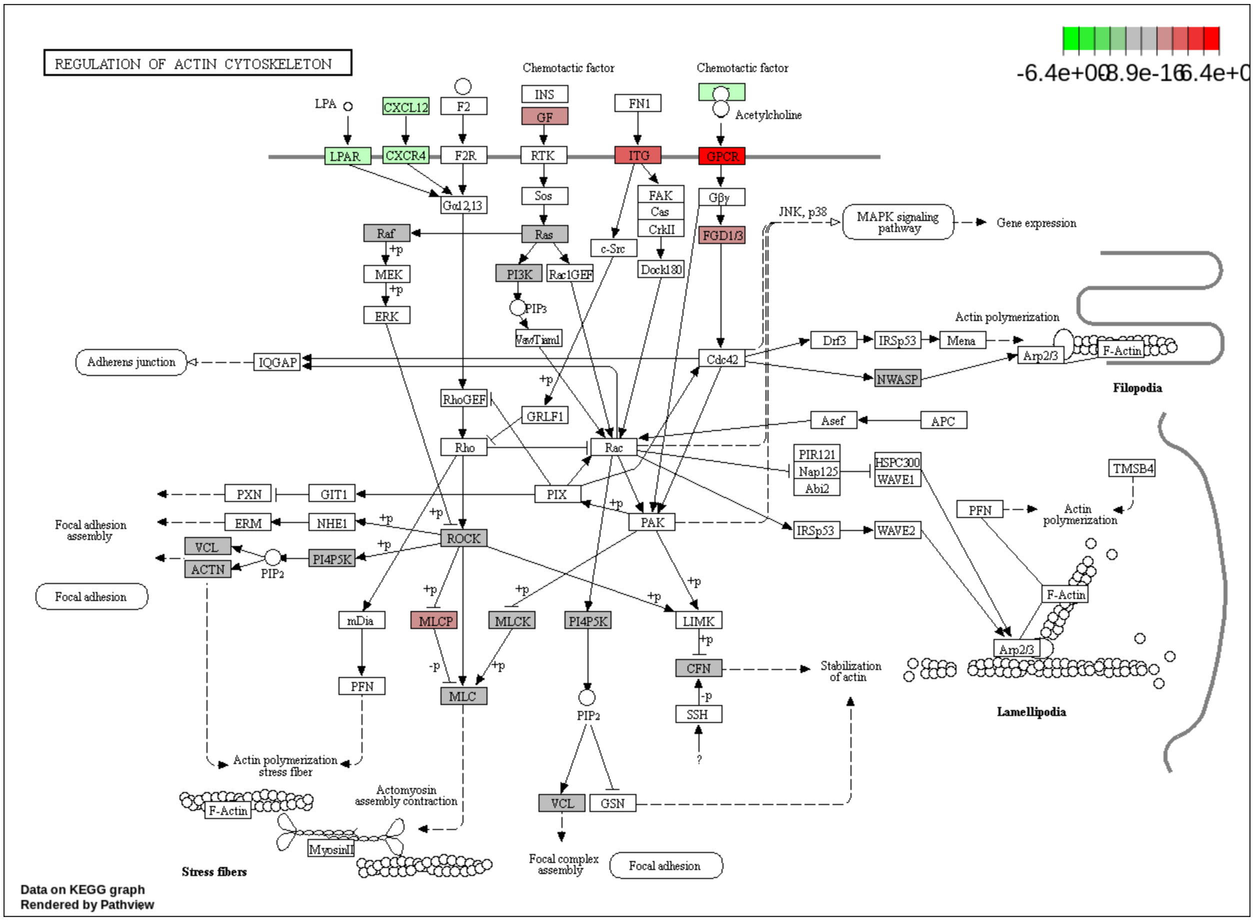

Supplementary Figure 12. Regulation of actin cytoskeleton alterations in etanercept-treated versus control NOD prostate. KEGG enrichment analysis highlighted significant changes of a set of genes within the regulation of actin cytoskeleton pathway. Downregulated genes are highlighted in green and upregulated genes are highlighted in red, where a greater intensity of color indicates a higher degree of change. Units indicate standardized fold-change.

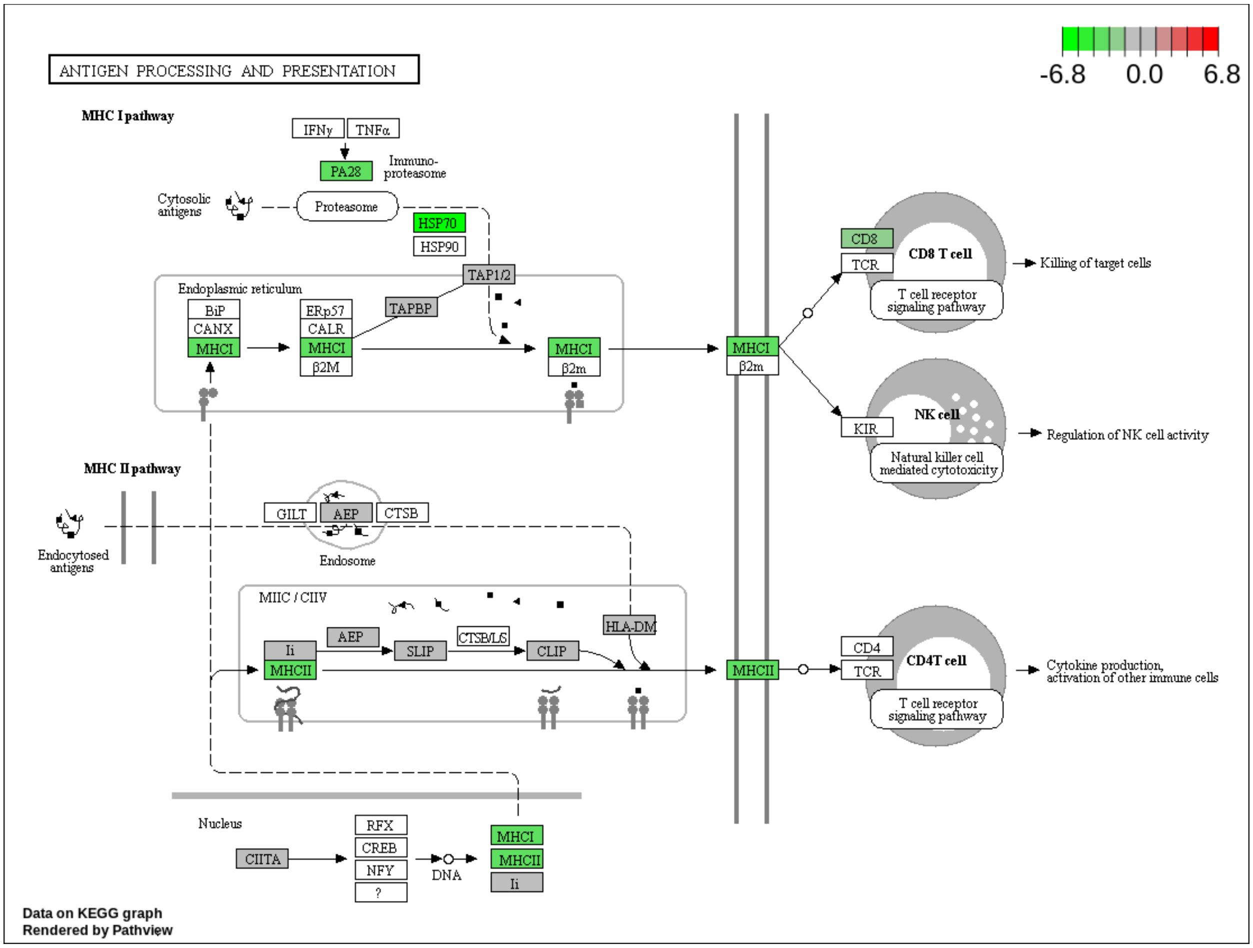

Supplementary Figure 13. Antigen processing and presentation alterations in etanercept-treated versus control NOD prostate. KEGG enrichment analysis highlighted downregulation of a set of genes within the antigen processing and presentation pathway. Downregulated genes are highlighted in green, where a greater intensity of color indicates greater downregulation. Units indicate standardized fold-change.

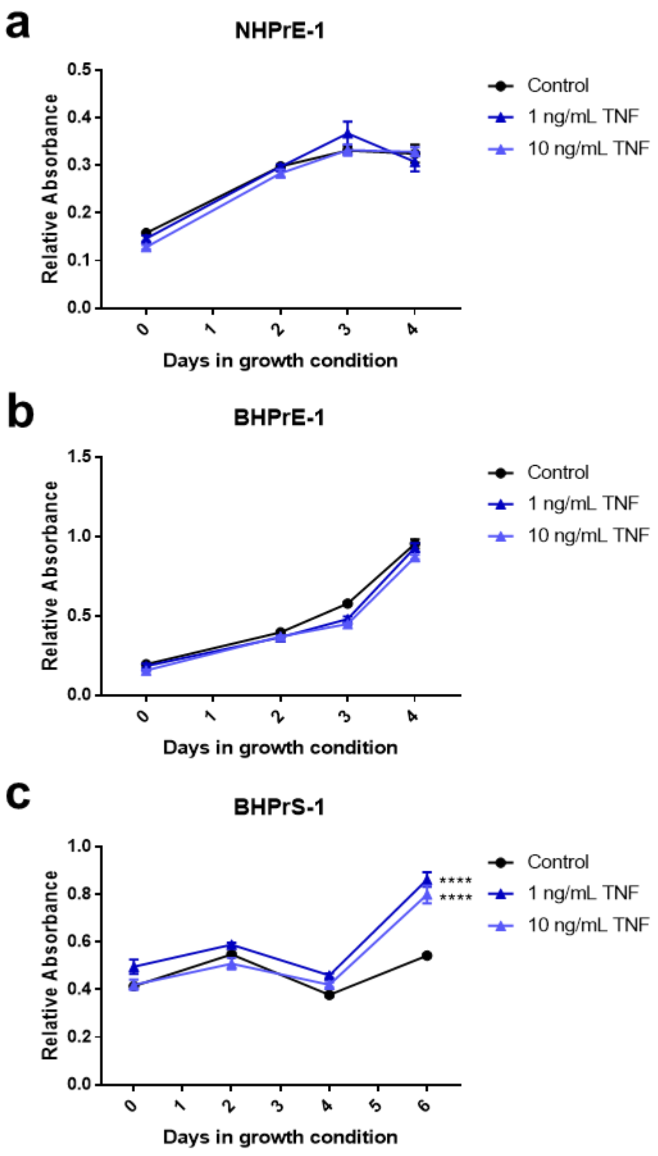

Supplementary Figure 14. TNF promotes stromal, but not epithelial, cell growth. Crystal violet growth assays were performed in low serum conditions (0.5%) with indicated treatments. **a)** NHPrE-1, **b)** BHPrE-1, and **c)** BHPrS-1 were grown in the presence or absence of 1 or 10 ng/mL recombinant TNF (dark and light blue, respectively), followed by fixation for crystal violet growth assay at indicated times. Points indicate the mean +/- SEM of at least five technical replicates and graphs are representative of n=3 independent experiments. Statistical analysis was performed

using a two-way ANOVA with Tukey’s multiple comparisons test, where **** represents $p<0.0001$ compared to control (black points). Source data are provided as a Source Data file.

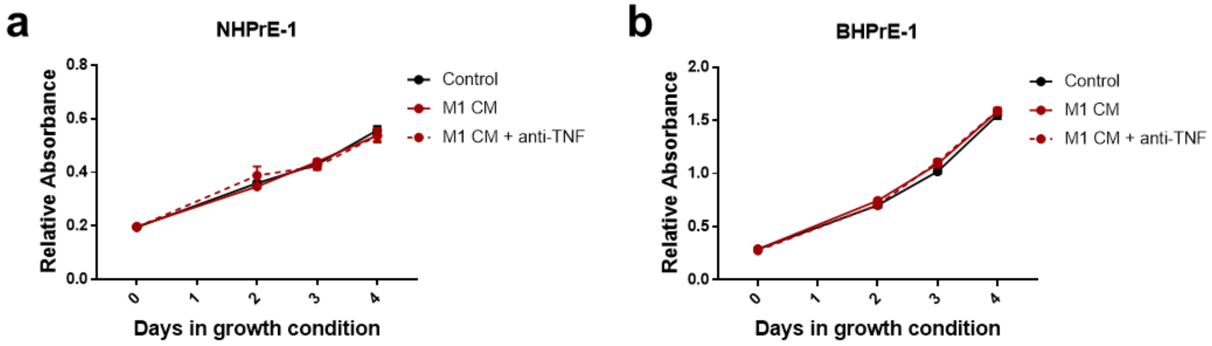

Supplementary Figure 15. Macrophage conditioned medium with or without TNF neutralization does not affect epithelial cell proliferation. Crystal violet growth assays were performed in low serum conditions (0.5%) for epithelial cell lines. **a)** NHPrE-1 and **b)** BHPrE-1 cells were grown in the presence of 50% M1 macrophage conditioned medium (red lines; generated from THP-1 cells) +/- 40 μg/mL TNF neutralizing antibody. The condition with anti-TNF neutralizing antibody is indicated with dashed lines. Points indicate the mean +/- SEM of six replicate wells, and graphs are representative of three independent experiments. Source data are provided as a Source Data file.

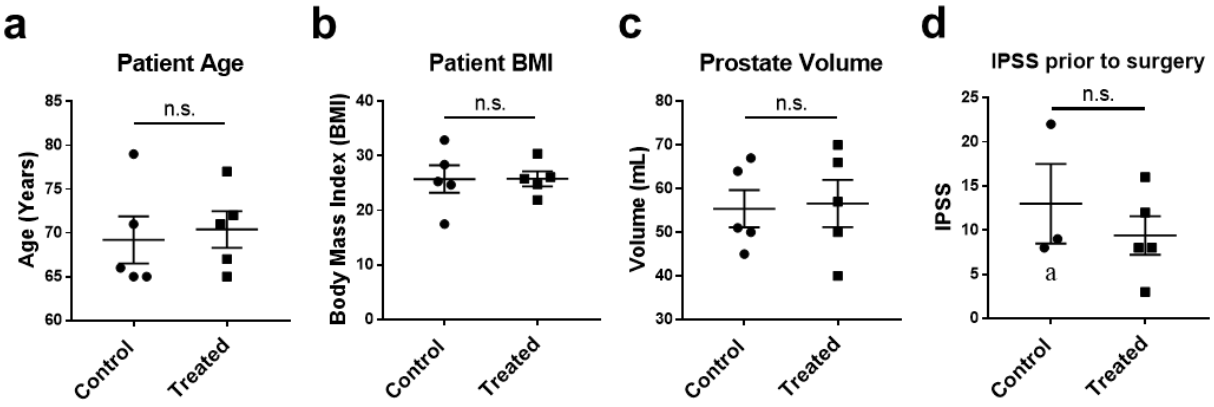

Supplementary Figure 16. Characteristics of TNF-antagonist treated or control patients. a) Patient age, **b)** patient BMI, **c)** prostate volume, and **d)** IPSS are shown for control patients (n=5 independent patients) and patients who were treated with TNF-antagonists (n=5 independent patients), from which the relevant IHC data was generated. Statistical analysis was completed using a two-tailed t-test and error bars indicate the mean +/- SEM for all graphs. Source data are provided as a Source Data file. n.s. = not significant. [a]two patients within the control group did not complete the IPSS questionnaire, thus have no values to report.

Supplementary Table 1. Diagnosis codes used for EDW study.

Diagnosis	ICD9 Codes	ICD10 Codes
Benign prostatic hyperplasia	600.x	N40.x
Prostate cancer	185	C61
Rheumatoid Arthritis	714.x	M05.x; M06.x; M08.0
Psoriasis	696.x	L40.x; M07.3; M09.0
Crohn’s Disease	555.x	K50.x; M07.4; M09.1
Ulcerative Colitis	556.x	K51.x; M07.5; M09.2
Multiple Sclerosis	340	G35
Vasculitis	446.x	L95.x; M30.x; M31.x
Lupus	710; 695.4	M32.x; L93.x
Myasthenia Gravis	358	G70.x
Ankylosing Spondylitis	720	M45.x; M08.1
Celiac Disease	579	K90.0
Type 1 Diabetes Mellitus	250.01	E10.x
Graves	242	E05.0
Hashimoto’s Thyroiditis	245.2	E06.3
Other Autoimmune Diseases	279.4; 279.41; 279.49; 446.21; 704.01; 289.81; 283.0; 571.42; 356.0; 356.8; 258.1; 136.1; 710.3; 287.31; 357.0; 281.0; 710.4; 571.6; 710.2; 341.2; 446.4	-

Supplementary Table 2. Procedure codes used for EDW study.

Procedure Category	CPT Codes
Transurethral resection (e.g., transurethral resection of prostate)	52601; 52630; 52450; 52500; 52510; 52612; 52614
Laser (e.g., greenlight photovaporization of prostate)	52647; 52648; 52649
Simple prostatectomy	55801; 55831; 55821
Prostatic urethral lift	52441; 52442

Supplementary Table 3. Medications used for EDW study.

Medication Category	Medication Name
Anti-TNF antibody	Infliximab
	Etanercept
	Adalimumab
	Golimumab
	Certolizumab
Selective costimulation modifier	Abatacept
Anti-IL-12 and Anti-IL-23 antibody	Ustekinumab
Antimetabolite	Methotrexate
Alpha-blocker	Doxazosin
	Terazosin
	Alfuzosin
	Tamsulosin
	Silodosin
5-alpha-reductase inhibitor	Finasteride
	Dutasteride
Beta-3-agonist	Mirabegron

Supplementary Table 4. Breakdown of patient age at the time of EDW data collection.

Patient Age (at data collection):	Frequency	Percent
40-49	26577	23.70
50-59	30260	26.98
60-69	26226	23.38
70-79	16064	14.32
80-89	9682	8.63
90+	3343	2.98

Supplementary Table 5. Breakdown of patient race in EDW.

Patient Race:	Frequency	Percent
African American	4473	3.99
American Indian/Alaska Native	268	0.24
Asian	4073	3.63
Caucasian	75919	67.71
Declined/Unknown	14	0.01
Other	27307	24.35
Pacific Islander/Hawaiian Native	67	0.06
Missing	31	

Supplementary Table 6. Breakdown of patient ethnicity in EDW.

Patient Ethnicity:	Frequency	Percent
Declined/Unknown	20	0.02
Hispanic/Latino	4166	3.72
Non-Hispanic	107933	96.27
Missing	33	

Supplementary Table 7. Proportion of men with BPH by autoimmune disease status. Two-sided chi-square tests were utilized to compare the proportion of BPH diagnoses in men with an autoimmune (AI) condition versus men with no AI condition. One compares men with any diagnosis of an AI condition to men without, while the second comparison includes only men diagnosed with an AI condition prior to their clinical BPH diagnosis, as a subset of patients who may have been treated for their AI condition (9,274 AI patients were diagnosed with AI disease prior to a BPH diagnosis out of 10,769 total patients with AI disease). Subcategories of the AI disease population are provided. The bolded p-values indicate a significant difference in BPH prevalence compared to the "no autoimmune disease" group. [a]indicates a significantly higher BPH prevalence than the 20.3% reference, although the incidence rate in this subpopulation of RA patients is decreased from 38.0% prevalence in all RA patients. [b]significant p-values are bolded.

Diagnosis of Men	Patients (%)	BPH Prevalence (N)	P-Value
No Autoimmune Disease	101,383 (90.4%)	20.3% (20,586)	Reference
Autoimmune Disease (all)	10,769 (9.6%)	30.6% (3,294)	**<0.001**
• Psoriasis	3,215 (2.9)	24.9%	**<0.001**
• Rheumatoid Arthritis	1,700 (1.5)	38.0%	**<0.001**
• Ulcerative Colitis	1,104 (1.0)	30.2%	**<0.001**
• Type 1 Diabetes	1,043 (0.9)	32.0%	**<0.001**
• Crohn's Disease	971 (0.9)	27.4%	**<0.001**
• Multiple Sclerosis	375 (0.3)	21.6%	0.884
• Celiac Disease	373 (0.3)	24.4%	0.142
• Hashimoto's Thyroiditis	372 (0.3)	22.9%	0.463
• Ankylosing Spondylitis	287 (0.3)	25.4%	0.086
• Lupus	137 (0.1)	30.7%	**0.007**
• Vasculitis	47		
• Myasthenia Gravis	32		
• Graves' Disease	3		
• Other	2,613 (2.3)		
Autoimmune Disease (prior to BPH diagnosis)	9,274 of total 10,769	19.4% (1,799)	**0.037**
• Psoriasis		16.3%	**<0.001**
• Rheumatoid Arthritis		23.2%[a]	**0.007**
• Ulcerative Colitis		20.7%	0.773
• Type 1 Diabetes		19.4%	0.521
• Crohn's Disease		19.4%	0.521
• Multiple Sclerosis		15.8%	**0.035**
• Celiac Disease		13.5%	**0.002**
• Hashimoto's Thyroiditis		11.4%	**<0.001**
• Ankylosing Spondylitis		18.0%	0.357
• Lupus		16.7%	0.334

Supplementary Table 8. Medication use in men treated for an autoimmune condition prior to BPH diagnosis (n=1,024).

AI Diseases/Drug	Total N (%)
All AI Diseases	1,024
• Methotrexate only	319 (31.2)
• TNF-antagonist only	485 (47.4)
• Both Methotrexate and TNF	211 (20.6)
• Others (T cell and Interleukin)	9 (0.8)
Psoriasis	465
• Methotrexate only	113 (24.3)
• TNF-antagonist only	235 (50.5)
• Both Methotrexate and TNF	109 (23.5)
• Others (T cell and Interleukin)	8 (1.7)
Rheumatoid Arthritis	415
• Methotrexate only	199 (48.0)
• TNF-antagonist only	89 (21.5)
• Both Methotrexate and TNF	125 (30.0)
• Others (T cell and Interleukin)	2 (0.5)
Ulcerative Colitis	106
• Methotrexate only	7 (6.6)
• TNF-antagonist only	89 (84.0)
• Both Methotrexate and TNF	10 (9.4)
• Others (T cell and Interleukin)	0
Type 1 Diabetes	13
• Methotrexate only	6 (46.2)
• TNF-antagonist only	5 (38.5)
• Both Methotrexate and TNF	2 (15.3)
• Others (T cell and Interleukin)	0
Crohn’s Disease	163
• Methotrexate only	12 (7.4)
• TNF-antagonist only	135 (82.8)
• Both Methotrexate and TNF	16 (9.8)
• Others (T cell and Interleukin)	0
Multiple Sclerosis	3
• Methotrexate only	3 (100)
• TNF-antagonist only	0
• Both Methotrexate and TNF	0
• Others (T cell and Interleukin)	0
Celiac Disease	8
• Methotrexate only	1 (12.5)
• TNF-antagonist only	4 (50.0)
• Both Methotrexate and TNF	3 (37.5)
• Others (T cell and Interleukin)	0
Hashimoto’s Thyroiditis	5
• Methotrexate only	4 (80.0)
• TNF-antagonist only	0
• Both Methotrexate and TNF	1 (20.0)
• Others (T cell and Interleukin)	0
Ankylosing Spondylitis	77
• Methotrexate only	7 (9.1)
• TNF-antagonist only	59 (76.6)
• Both Methotrexate and TNF	11 (14.3)
• Others (T cell and Interleukin)	0
Lupus	14
• Methotrexate only	8 (57.1)
• TNF-antagonist only	5 (35.7)
• Both Methotrexate and TNF	1 (7.2)
• Others (T cell and Interleukin)	0

Supplementary Table 9. BPH diagnosis rate in autoimmune disease patients based on medication use. Two-sided chi-square or Fisher exact tests were used to compare the rate of BPH diagnoses in men with an AI condition who were treated or not with medication. The p-values in column [a] are compared to the reference BPH rate of patients without a diagnosed AI disease, and the p-values in column [b] are compared to the BPH rate of patients not treated for each AI disease. [c]significant p-values are bolded.

AI Diseases/Drug	Total N	BPH Rate (%)	P-value[a]	P-value[b]
Patients without AI disease	101,383	20.3	Reference	
Patients with AI disease prior to BPH diagnosis	9274	19.4	**0.037**	
All AI Disease Patients treated with medication	1024	21.1	0.347	
• Methotrexate only	319	31.0	**<0.0001**	**<0.0001**
• TNF-antagonists only	485	15.3	**0.006**	**0.0314**
AI patients without medication	8250	19.2	**0.017**	Reference
Psoriasis				
• Methotrexate only	113	25.7	0.157	**0.0282**
• TNF-antagonists only	235	14.5	**0.026**	0.4795
• No medication	404	16.6	0.064	Reference
Rheumatoid Arthritis				
• Methotrexate only	199	36.2	**<0.0001**	**0.0044**
• TNF only	89	28.1	0.068	0.419
• No medication	240	23.9	0.184	Reference
Ulcerative Colitis				
• Methotrexate only	7	42.9	0.138	0.3942
• TNF-antagonists only	89	19.1	0.779	0.6009
• No medication	193	21.9	0.614	Reference
Type 1 Diabetes				
• Methotrexate only	6	66.7	**0.021**	**0.0285**
• TNF-antagonists only	5	20.0	1.000	0.9748
• No medication	181	20.5	0.964	Reference
Crohn's Disease				
• Methotrexate only	12	8.3	0.303	0.4608
• TNF-antagonists only	135	17.0	0.346	0.3078
• No medication	156	21.5	0.643	Reference
Multiple Sclerosis				
• Methotrexate only	3	66.7	0.201	0.1003
• TNF-antagonists only	0	-	-	-
• No medication	55	15.8	0.199	Reference
Celiac Disease				
• Methotrexate only	1	100.0	0.460	0.375
• TNF-antagonists only	4	50.0	0.393	0.2899
• No medication	46	14.2	0.392	Reference
Hashimoto's Thyroiditis				
• Methotrexate only	4	0.0	0.699	0.9383
• TNF-antagonists only	0	-	-	-
• No medication	40	12.4	0.220	Reference
Ankylosing Spondylitis				
• Methotrexate only	7	28.6	0.938	0.8875
• TNF-antagonists only	59	18.6	0.752	0.9058
• No medication	34	18.2	0.700	Reference
Lupus				
• Methotrexate only	8	37.5	0.441	0.5604
• TNF-antagonists only	5	40.0	0.590	0.6588
• No medication	17	16.5	0.975	Reference

Supplementary Table 10. Risk of ulcerative colitis diagnosis after medication use in patients with other AI diseases (n=9,274). Two-sided Fisher's exact test was used to evaluate the rate of ulcerative colitis diagnosis after the indicated treatments, followed by post-hoc analysis using Bonferroni correction for multiple testing (p<0.0001). Percent of patients in parentheses indicates the percent of patients with the indicated drug treatment in each row. [a]significant p-values are bolded.

Treatment	Patients with AI disease other than ulcerative colitis N (%)	Patients with ulcerative colitis diagnosis prior to or at time of prescribing medication N (%)	Ulcerative colitis diagnosis after taking medication N (%)	P-value	Post-hoc test	P-value
Methotrexate only	312 (97.8)	0 (0)	7 (2.2)	**<0.0001**	TNF-antag. only vs. Methotrexate	**<0.0001**
TNF-antagonists only	396 (81.7)	67 (13.8)	22 (4.54)		Reference	
Both Methotrexate and TNF-antagonists	201 (95.3)	5 (2.4)	5 (2.4)		TNF-antag. vs. Both	**<0.0001**
Others (T cell /Interleukin)	9 (100)	0 (0)	0 (0)		N/A	
No medication	7368 (89.3)	0 (0)	882 (10.7)		TNF-antag. only vs. No medication	**<0.0001**

Supplementary Table 11. Risk of Crohn's disease diagnosis after medication use in patients with other AI diseases (n=9,274). Two-sided Fisher's exact test was used to evaluate the rate of Crohn's disease diagnosis after the indicated treatments, followed by post-hoc analysis using Bonferroni correction for multiple testing. Percent of patients in parentheses indicates the percent of patients with the indicated drug treatment in each row. [a]significant p-values are bolded.

Treatment	Patients with AI disease other than Crohn's N (%)	Patients with Crohn's diagnosis prior to or at time of prescribing medication N (%)	Crohn's diagnosis after taking medication N (%)	P-value	Post-hoc test	P-value
Methotrexate only	318 (99.7)	0 (0)	1 (0.3)	**<0.0001**	TNF-antag. vs. Methotrexate	0.6504
TNF-antagonists only	481 (99.2)	3 (0.6)	1 (0.2)		Reference	
Both Methotrexate and TNF-antagonists	208 (98.6)	0 (0)	3 (1.4)		TNF-antag. vs. Both	0.3284
Others (T cell /Interleukin)	9 (100)	0 (0)	0 (0)		N/A	
No medication	7925 (96.1)	0 (0)	325 (3.9)		TNF-antag. vs. No medication	**<0.0001**

Supplementary Table 12. Most significantly altered pathways in CD45$^+$ cells from large versus small human BPH tissues. Ingenuity Pathway Analysis (Qiagen) was conducted using the differentially expressed genes between large versus small CD45$^+$ scRNA-seq samples. A one-tailed Fisher's exact test was used to determine pathway enrichment, and the Benjamini-Hochberg procedure was used to control for multiple hypothesis testing. [a]gray highlighted rows indicate pathways related to AI diseases.

Ingenuity Canonical Pathways	-Log(p-value)	Ratio
Communication between Innate and Adaptive Immune Cells	11.5	0.177
Hematopoiesis from Pluripotent Stem Cells	10	0.245
EIF2 Signaling	7.94	0.0893
NRF2-mediated Oxidative Stress Response	7.64	0.0952
Systemic Lupus Erythematosus In B Cell Signaling Pathway	7.13	0.0764
Atherosclerosis Signaling	6.91	0.111
B Cell Receptor Signaling	6.29	0.0865
Role of Macrophages, Fibroblasts and Endothelial Cells in Rheumatoid Arthritis	6.21	0.0673
IL-10 Signaling	6.17	0.145
IL-6 Signaling	6.12	0.104
Dendritic Cell Maturation	5.64	0.082
Primary Immunodeficiency Signaling	5.38	0.16
Systemic Lupus Erythematosus Signaling	5.08	0.0699
Granulocyte Adhesion and Diapedesis	5.04	0.0778
Cholecystokinin/Gastrin-mediated Signaling	4.78	0.0924
Graft-versus-Host Disease Signaling	4.5	0.146
Autoimmune Thyroid Disease Signaling	4.44	0.143
Allograft Rejection Signaling	4.44	0.105

Supplementary Table 13. Antibodies used for all studies. Table indicates the antibody target, clone (if monoclonal), manufacturer product number, and dilution used for all antibodies used for all studies in prostate tissues.

Application	Tissue Sample	Antibody Target	Clone	Species	Isotype	Company	Ref. #	Antibody Dilution
IHC	Human	phospho-NFkB p65 (S276)	polyclonal	Rabbit	IgG	Abcam	ab194726	1:100
IHC	Human	Ki67	polyclonal	Rabbit	IgG	Abcam	ab15580	1:50
IHC	Human	CD68	EPR20545	Rabbit	IgG	Abcam	ab213363	1:100
IHC	NOD	F4/80	polyclonal	Rabbit	IgG	Abcam	ab100790	1:100
IHC	NOD	Ki67	polyclonal	Rabbit	IgG	Abcam	ab15580	1:75
IHC	NOD	phospho-NFkB p65 (S276)	polyclonal	Rabbit	IgG	Abcam	ab194726	1:100
IHC	PB-PRL	F4/80	polyclonal	Rabbit	IgG	Abcam	ab100790	1:100
IHC	PB-PRL	Ki67	polyclonal	Rabbit	IgG	Abcam	ab15580	1:200
IHC	PB-PRL	phospho-NFkB p65 (S276)	polyclonal	Rabbit	IgG	Abcam	ab194726	1:100
FACS	Human	CD45-PE	HI30	Mouse	IgG1,k	Biolegend	304058	1:20
FACS	Human	EpCAM-APC	9C4	Mouse	IgG2b,k	Biolegend	324208	1:20
FACS	Human	CD200-PE/Cy7	OX-104	Mouse	IgG1,k	Biolegend	329212	1:20
Flow	Human	CD45-FITC	HI30	Mouse	IgG1,k	Biolegend	304006	1:20
Flow	Human	CD11b-PE/Cy7	ICRF44	Mouse	IgG1,k	Biolegend	301322	1:20
Flow	Human	CD19-APC/Cy7	HIB19	Mouse	IgG1,k	Biolegend	302218	1:20
Flow	Human	CD3-APC	UCHT1	Mouse	IgG1,k	Biolegend	300412	1:20
Flow	Human	CD4-PE	RPA-T4	Mouse	IgG1,k	Biolegend	300508	1:20
Flow	Human	CD8-BV510	RPA-T8	Mouse	IgG1,k	Biolegend	301048	1:20
CITE-seq	Human	CD3	UCHT1	Mouse	IgG1,k	Biolegend	300477	1:50
CITE-seq	Human	CD4	RPA-T4	Mouse	IgG1,k	Biolegend	300565	1:50
CITE-seq	Human	CD8	RPA-T8	Mouse	IgG1,k	Biolegend	301069	1:50
CITE-seq	Human	CD11b	ICRF44	Mouse	IgG1,k	Biolegend	301357	1:50
CITE-seq	Human	CD19	HIB19	Mouse	IgG1,k	Biolegend	302263	1:50
ELISA	Human	Anti-etanercept	ETA63C8	Mouse	IgG1,k	Millipore	MABF1973	0-500 ng/mL
ELISA	Human	Anti-mouse IgG	polyclonal	Horse	IgG	Cell Signaling	7076	1:10,000
Neutralization	Human	TNF	polyclonal	Rabbit	IgG	Fisher	P300A	40 µg/mL

Supplementary Table 14. Sequencing metrics for scRNA-seq of BPH associated CD45⁺ leukocytes. Table includes the run metrics from each scRNA-seq sample, including 10 small and 4 large samples. The metrics indicate high quality data and that the average number of cells captured approached the intended number of 5000 cells. The final row indicates the average indicated metric for all samples.

Sample	Number of Reads	% Q30 Bases in Read	% Reads Mapped	Estimated Number of Cells	Mean Reads per cell	Median Genes per cell
003_small	437,303,375	91.3	96.2	6,388	68,457	1692
004_small	266,767,655	91.7	96.7	4,163	64,080	1599
006_small	258,138,447	91.5	96.9	6,427	40,164	1628
007_small	765,500,750	90.8	96.0	4,427	172,916	1958
008_small	552,744,091	93.0	98.1	5,053	109,389	1652
009_small	361,879,675	93.3	96.7	3,659	98,901	1740
010_small	245,628,503	91.6	96.3	3,750	65,500	1576
012_large	297,618,482	90.2	96.7	5,826	51,084	1526
013_small	393,520,430	92.6	96.7	5,230	75,242	1476
1144_small	394,479,925	90.0	97.0	6,010	65,637	1662
1157_large	303,893,852	90.4	96.8	6,816	44,585	1525
1195_large	455,027,712	92.4	96.6	5,377	84,624	1591
1196_small	224,350,221	92.7	96.8	4,554	49,264	1489
766_large	368,324,247	90.8	95.6	4,076	90,364	1335
AVERAGE:	380,369,812	91.6	96.7	5,125	77,158	1,604

Supplementary Table 15. Sequencing metrics for scRNA-seq of BPH associated cells. Table includes the run metrics from five scRNA-seq samples, each derived from a prostate transition zone tissue after simple prostatectomy. The metrics indicate high quality data and that the average number of cells captured exceeded the intended number of 10,000 cells. The final row indicates the average indicated metric for all samples.

Sample	Number of Reads	% Q30 Bases in Read	% Reads Mapped	Estimated Number of Cells	Mean Reads per cell	Median Genes per cell
1562_all	548,579,606	91.4	95.6	22,486	24,396	1,084
1579_all	712,308,756	92.0	96.5	13,132	54,242	2,518
1595_all	710,365,797	91.5	96.8	11,687	60,783	2,326
1628_all	654,692,830	90.9	94.7	10,090	64,885	1,650
1652_all	832,511,485	91.5	96.4	10,507	79,234	2,299
AVERAGE:	691,691,695	91.5	96	13,580	56,708	1,975
