# Supplementary material for: TNF is a potential therapeutic target to suppress prostatic inflammation and hyperplasia in autoimmune disease
Source: Nat Commun. 2022 Apr 19;13:2133. doi: 10.1038/s41467-022-29719-1 (PMC9018703; doi:10.1038/s41467-022-29719-1)
Supplement: Supplementary file 3 — Description of Additional Supplementary Files [file 41467_2022_29719_MOESM3_ESM.pdf]

Description of Additional Supplementary Files

Supplementary Data 1.

Combinations of AI diseases in patients with an AI disease diagnosis prior to BPH diagnosis (n=9,274). The number of patients with each combination of AI diseases are listed.
