# Supplementary material for: TNF is a potential therapeutic target to suppress prostatic inflammation and hyperplasia in autoimmune disease
Source: Nat Commun. 2022 Apr 19;13:2133. doi: 10.1038/s41467-022-29719-1 (PMC9018703; doi:10.1038/s41467-022-29719-1)
Supplement: Supplementary file 5 — Reporting Summary [file 41467_2022_29719_MOESM5_ESM.pdf]

nature research

Corresponding author(s): Simon W. Hayward, PhD

Last updated by author(s): February 17, 2022

Reporting Summary

Nature Research wishes to improve the reproducibility of the work that we publish. This form provides structure for consistency and transparency in reporting. For further information on Nature Research policies, see our Editorial Policies and the Editorial Policy Checklist.

Statistics

For all statistical analyses, confirm that the following items are present in the figure legend, table legend, main text, or Methods section.

n/a	Confirmed	
☐	☒	The exact sample size (n) for each experimental group/condition, given as a discrete number and unit of measurement
☐	☒	A statement on whether measurements were taken from distinct samples or whether the same sample was measured repeatedly
☐	☒	The statistical test(s) used AND whether they are one- or two-sided<br>*Only common tests should be described solely by name; describe more complex techniques in the Methods section.*
☐	☒	A description of all covariates tested
☐	☒	A description of any assumptions or corrections, such as tests of normality and adjustment for multiple comparisons
☐	☒	A full description of the statistical parameters including central tendency (e.g. means) or other basic estimates (e.g. regression coefficient) AND variation (e.g. standard deviation) or associated estimates of uncertainty (e.g. confidence intervals)
☐	☒	For null hypothesis testing, the test statistic (e.g. F, t, r) with confidence intervals, effect sizes, degrees of freedom and P value noted<br>*Give P values as exact values whenever suitable.*
☒	☐	For Bayesian analysis, information on the choice of priors and Markov chain Monte Carlo settings
☒	☐	For hierarchical and complex designs, identification of the appropriate level for tests and full reporting of outcomes
☐	☒	Estimates of effect sizes (e.g. Cohen's d, Pearson's r), indicating how they were calculated

Our web collection on statistics for biologists contains articles on many of the points above.

Software and code

Policy information about availability of computer code

Data collection	Enterprise Database Warehouse study: Patient data were collected via enterprise database warehouse (EDW) as well as Excel (2016 edition).<br>scRNA-seq study: Sequencing reads from the Chromium system were de-multiplexed and processed using the CellRanger pipeline v3.0.0 (10x Genomics). CellRanger mkfastq v3.0.0 was run to generate FASTQ files where dual indices were ignored, barcode mismatch allowance was set to 0, and the flag "—use-bases-mask=Y26n*,I8n*,n*,Y98n" was set.
Data analysis	scRNA-seq study: CellRanger count v3.0.0 was then used for alignment, filtering, barcode counting, and unique molecular identifier (UMI) counting. All reads were aligned to the ENSEMBL human genome version GrCh38 using the STAR aligner v2.5.4. Seurat version 3.1.3 was used for data normalization and cell clustering based on differential gene expression. Within the Seurat analysis, a resolution of 0.2 was used in detecting communities of cells via the Louvain algorithm, which is included in Seurat through the package Modularity Optimizer version 1.3.0 and is called via the FindClusters() function. The R package clustree version 0.4.3 was used to determine optimal resolution to use in clustering. Data were normalized using scTransform v.0.3.1 and cell cycle-related genes were used to produce a cell cycle score for each cell. Cell cycle scores, mitochondrial reads, and UMI counts were used to regress out heterogeneity from these variables by scaling the data. R version 3.5.1 was used for scRNA-seq statistical analyses. Bioconductor version 3.8 and R v. 3.5.1 were used for all scRNA-seq analyses. Differentially expressed genes between small and large sample groups were identified using the edgeR Bioconductor package, v 3.31 with an FDR cutoff of 5%. A stable version of the R scripts used to perform the single-cell RNA-seq analysis are available at https://github.com/natallah/BPH_scRNAseq_NatureComm.git and Zenodo at https://doi.org/10.5281/zenodo.5484422 through an Apache 2.0 license, allowing users to freely use the scripts for any purpose.<br><br>Bulk RNA-seq: For bulk RNA-seq analysis, mapping to the reference genome was performed with STAR aligner version 2.6.1d, allowing 2 mismatches. Quantification of reads mapping to genes was performed with FeatureCounts version 1.5.0-p3 using default parameters. DESeq2 version 1.20.0 was used to perform a differential expression analysis and p-values were adjusted for multiple testing using the Benjamini-Hochberg method. Significantly differentially expressed genes were determined based on an adjusted p-value≤0.05. ClusterProfiler version 3.8.1 was used for enrichment analyses. Kyoto Encyclopedia of Genes and Genomes (KEGG) was used for enrichment analysis and

visualization of altered pathways.

Flow Cytometry: BD FACS Diva v6.1.3 and FlowLogic v7 (Miltenyi) were used for flow cytometry analysis.

All other studies: Data were analyzed using Prism software version 7.05 or version 8 (GraphPad) and SAS 9.4 version (Cary, NC).

For manuscripts utilizing custom algorithms or software that are central to the research but not yet described in published literature, software must be made available to editors and reviewers. We strongly encourage code deposition in a community repository (e.g. GitHub). See the Nature Research guidelines for submitting code & software for further information.

Data

Policy information about availability of data

All manuscripts must include a data availability statement. This statement should provide the following information, where applicable:

- Accession codes, unique identifiers, or web links for publicly available datasets
- A list of figures that have associated raw data
- A description of any restrictions on data availability

The scRNA-seq data is available in GEO under accession numbers GSE164695 (leukocytes) [https://www.ncbi.nlm.nih.gov/geo/query/acc.cgi?acc=GSE164695] and GSE183676 (all cells) [https://www.ncbi.nlm.nih.gov/geo/query/acc.cgi?acc=GSE183676]. The bulk RNA-seq of NOD prostate tissues is available in GEO under accession number GSE183414 [https://www.ncbi.nlm.nih.gov/geo/query/acc.cgi?acc=GSE183414]. The raw EDW data is not available due to IRB-restrictions. Source data are provided with this paper. Any further information about tissue resources and reagents associated with these studies should be directed to, and will be fulfilled by, the corresponding author upon reasonable request.

Field-specific reporting

Please select the one below that is the best fit for your research. If you are not sure, read the appropriate sections before making your selection.

[x] Life sciences [ ] Behavioural & social sciences [ ] Ecological, evolutionary & environmental sciences

For a reference copy of the document with all sections, see nature.com/documents/nr-reporting-summary-flat.pdf

Life sciences study design

All studies must disclose on these points even when the disclosure is negative.

Sample size

For the EDW study, sample size was determined with adequate power and a retrospective evaluation of all patients between 01/01/2010 and 12/31/2012 in our health system were included. The 14 male tissues evaluated by scRNA-seq were collected over approximately 8 months time; analysis of additional tissues is ongoing in our laboratory. For animal studies, initial power calculations indicated a need for six mice per group. At the p< 0.05 level this group size provides 80% power to detect a difference in means of 1.80 standardized units. To account for the high mortality and variable phenotypes produced in NOD mice we increased the initial group size to ensure sufficient samples for analysis by the time the animals were euthanized. Post hoc analysis of individual experiments was performed to determine statistical significance of data as described for individual outcomes. The human prostate tissues from patients treated with TNFa-antagonists is limited by the number of patients in our biorepository (started in 2015). scRNA-seq samples were limited by the number of large prostate tissues (more than 90 grams). The sample size for bulk RNA-seq was limited by the number of samples stored frozen for downstream analysis (n=4 per group), but this sample size was adequate to identify significantly altered pathways between groups. For in vitro studies utilizing primary cell cultures, evaluation was limited based on the number of primary cultures isolated from simple prostatectomy tissues.

Data exclusions

EDW study: Patients under 40 years of age or with a diagnosis of prostate cancer were excluded from the study.
scRNA-seq studies: Patients with a diagnosis of prostatitis, in-dwelling catheter at the time of surgery, or cancer of Gleason score >7 were excluded from evaluation.
Animal studies: One animal in the Pb-PRL control group was eliminated from statistical analysis after 12 weeks treatment due to rapid prostate swelling, determined on necropsy to likely have arisen from a hemorrhage. However, this data point is still presented per reviewer request.
Human prostate tissues: Biorepository specimens that had very little tissue for analysis were excluded.

Replication

In experiments where data is displayed with technical replicates, such as in vitro studies, reproducibility was verified by conducting at least three independent experiments. Results are representative of the independent experiments.

Randomization

For the EDW study, men were assigned to groups based on disease diagnosis or treatment with specific therapeutic classes. For scRNA-seq studies, patients were sorted into categories based on pre-operative prostate size. For animal studies, mice were randomly assigned to groups using block stratification. Human tissues utilized for pathological evaluation of the prostate were assigned to groups based on a patient's therapeutic use of TNF-antagonists (or lack thereof). In all relevant studies, patient groups were either age- and BMI-matched or these covariates were controlled for via statistical methods. For example, part of the EDW study controlled for age, race, ethnicity, and BMI covariates.

Blinding

Data collection for the EDW study was an unbiased acquisition of medical record data limited to male patients over age 40 between dates 1/1/2010 - 12/31/2012, but blinding was not possible due to the need to assign groups for subsequent analysis. scRNA-seq and bulk RNA-seq studies require that a group identity is assigned for downstream analysis, but bioinformatic processing and analysis is identical regardless of group designation. Unsupervised clustering was performed in scRNA-seq analysis so group identity did not bias cell clustering. Pathological evaluation of human and mouse tissues was blinded. In vitro studies were not blinded due to the need for direct treatment of cells for growth assays, but all samples contained in individual graphs were processed identically.

Reporting for specific materials, systems and methods

We require information from authors about some types of materials, experimental systems and methods used in many studies. Here, indicate whether each material, system or method listed is relevant to your study. If you are not sure if a list item applies to your research, read the appropriate section before selecting a response.

Materials & experimental systems

n/a	Involved in the study
☐	☒ Antibodies
☐	☒ Eukaryotic cell lines
☒	☐ Palaeontology and archaeology
☐	☒ Animals and other organisms
☐	☒ Human research participants
☒	☐ Clinical data
☒	☐ Dual use research of concern

Methods

n/a	Involved in the study
☒	☐ ChIP-seq
☐	☒ Flow cytometry
☒	☐ MRI-based neuroimaging

Antibodies

Antibodies used

scRNA-seq FACS:
Zombie Violet, Biolegend 423114, lot B254627
CD45-PE [clone HI30], Biolegend 304058, lot B299749
EpCAM-APC [clone 9C4], Biolegend 324208, lot B284158
CD200-PE/Cy7 [clone OX-104], Biolegend 329212, lot B226001

Flow cytometry analysis:
Zombie Violet, Biolegend 423114, lot B254627
CD45-FITC [clone HI30], Biolegend 304006 lot B233140
CD11b-PE/Cy7 [clone ICRF44], Biolegend 301322, lot B247020
CD19-APC/Cy7 [clone HIB19], Biolegend 302218, lot B279663
CD3-APC [clone UCHT1], Biolegend 300412, lot B252651
CD4-PE [clone RPA-T4], Biolegend 300508, lot B266072
CD8-BV510 [clone RPA-T8], Biolegend 301048, lot B281246

IHC:
anti-Ki67, Abcam ab15580, lot GR3293897-2
anti-F4/80, Abcam ab100790, lot GR3229582-15
anti-phospho NFkB p65, Abcam ab194726, lot GR3371448-1
anti-CD68, Abcam ab213363, clone EPR20545, lot GR3266939-2

auto-antibody ELISA:
anti-etanercept antibody [clone ETA63C8], EMD Millipore MABF1973, dilution 0-500 ng/mL
HRP-conjugated anti-mouse IgG secondary, Cell Signaling 7076, dilution 1:10,000

Neutralization:
anti-human tumor necrosis factor, Fisher P300A, dilution 40ug/mL

CITE-seq (Total-seq antibodies):
anti-human CD3 [clone UCHT1], Biolegend 300477, dilution 1:50
anti-human CD4 [clone RPA-T4], Biolegend 300565, dilution 1:50
anti-human CD8 [clone RPA-T8], Biolegend 301069, dilution 1:50
anti-human CD11b [clone ICRF44], Biolegend 301357, dilution 1:50
anti-human CD19 [clone HIB19], Biolegend 302263, dilution 1:50

Validation

The antibodies used were commercially-validated.
For all Biolegend antibodies, the manufacturer indicates that "Each lot of this antibody is quality control tested by immunofluorescent staining with flow cytometry analysis."
CD45-PE and CD45-FITC: The product data sheet specifies human species reactivity.
EpCAM-APC: the product data sheet specifies human species reactivity.
CD200-PE/Cy7: The product data sheet specifies species reactivity to human, african green, and baboon.
CD11b-PE/Cy7 and anti-CD11b for CITE-seq: the product data sheet specifies human, african green, baboon, chimpanzee, common marmoset, cynomolgus, rhesus, and swine species reactivity.
CD19-APC/Cy7 and anti-CD19 for CITE-seq: the product data sheet specifies human, chimpanzee, and rhesus species reactivity.
CD3-APC and anti-CD3 for CITE-seq: The product data sheet specifies human species reactivity.
CD4-PE and anti-CD4 for CITE-seq: The product data sheet specifies human and chimpanzee species reactivity.

CD8-BV510 and anti-CD8 for CITE-seq: The product data sheet specifies human species reactivity.

anti-Ki67: From the manufacturer's website "Ab15580 is batch tested in ICC/IHC. A variability in IHC-Fr performance can occur with this antibody but we can guarantee consistency in IHC-P. The Life Science industry has been in the grips of a reproducibility crisis for a number of years. Abcam is leading the way in addressing this with our range of recombinant monoclonal antibodies and knockout edited cell lines for gold-standard validation." This product was validated for IHC-paraffin and with knockout cell lines. The product data sheet specifies human and mouse species reactivity.

anti-F4/80: From the datasheet "The Life Science industry has been in the grips of a reproducibility crisis for a number of years. Abcam is leading the way in addressing this with our range of recombinant monoclonal antibodies and knockout edited cell lines for gold-standard validation." This product was validated for IHC-paraffin. The product data sheet specifies human and mouse species reactivity.

anti-phospho NFkb p65: From the datasheet "The Life Science industry has been in the grips of a reproducibility crisis for a number of years. Abcam is leading the way in addressing this with our range of recombinant monoclonal antibodies and knockout edited cell lines for gold-standard validation." This product was validated for use in IHC-paraffin. The product data sheet specifies human and rat species reactivity.

anti-CD68: The manufacturer indicates in the datasheet that "this product is a recombinant monoclonal antibody, which offers several advantages including high batch-to-batch consistency and reproducibility, improved sensitivity and specificity, long-term security of supply, and animal-free production." This product was validated for IHC-paraffin staining. The product data sheet specifies human species reactivity.

anti-etanercept antibody: This antibody was validated by the manufacturer for ELISA and the data sheet indicates human species reactivity.

HRP-conjugated anti-mouse IgG secondary: The manufacturer's website indicates "This product is thoroughly validated with CST primary antibodies and will work optimally with the CST western immunoblotting protocol, ensuring accurate and reproducible results." The validated applications include ELISA-peptide, along with a variety of other assays. Human species reactivity is among the list of species for this antibody, although the manufacturer indicates that all species are expected to be valid for use of this secondary.

anti-human TNF, P300A antibody from Invitrogen was validated by the manufacturer for ELISA, IHC, western blot, and neutralization. The datasheet indicates reactivity with human samples.

Eukaryotic cell lines

Policy information about cell lines

Cell line source(s)	THP-1: ATCC<br>BHPrE-1, NHPrE-1, and BHPrS-1: produced in Hayward lab as referenced in the Methods section
Authentication	All cells have been authenticated by ATCC via STR profiling within 20 passages of testing.<br>THP-1: STRB0424<br>BHPrE-1: STRA3426<br>NHPrE-1: STRA3441<br>BHPrS-1: STRB0418
Mycoplasma contamination	BHPrE-1, NHPrE-1, and BHPrS-1 tested negative for mycoplasma using MycoAlert mycoplasma detection kit (Lonza). THP-1 cells were not tested for mycoplasma contamination.
Commonly misidentified lines<br>(See ICLAC register)	No commonly misidentified cell lines were used in these studies.

Animals and other organisms

Policy information about studies involving animals; ARRIVE guidelines recommended for reporting animal research

Laboratory animals	Male non-obese diabetic (NOD) mice were purchased from Jackson Laboratory (Bar Harbor, ME; Stock Number: 001976). Twenty total mice were treated with either drug or vehicle control for 5 weeks starting at 25 weeks of age. Male probasin-prolactin (Pb-PRL) transgenic mice on a C57Bl/6J background were from Dr. Kindblom, Sahlgrenska University Hospital. Eleven total Pb-PRL mice were treated with either drug or vehicle control for 12 weeks starting at 20-22 months of age. All animals were housed in facilities with an ambient room temperature of 21-22 degrees C, 30-70% relative humidity, and 12-hour light/dark cycles.
Wild animals	No wild animals were used in these studies.
Field-collected samples	No field-collected samples were used in these studies.
Ethics oversight	All NOD studies were conducted according to US federal and state regulations and approved by the NorthShore Institutional Animal Care and use Committee (IACUC; #EH-15-064). All Pb-PRL studies were performed in accordance with the National Institute of Health Guidelines for the Care and Use of Laboratory Animals and approved by the Roswell Park Institutional Animal Care and Use Committee (#1308M).

Note that full information on the approval of the study protocol must also be provided in the manuscript.

Human research participants

Policy information about studies involving human research participants

Population characteristics	As indicated above, patients under the age of 40 and men with a diagnosis of prostate cancer were excluded from the EDW study. Only male patients were evaluated with an age range of 40 to over 90 years of age. Age, BMI, and diagnosis of BPH and a range of autoimmune conditions were evaluated and detailed in supplementary data. Data that describe patient race, ethnicity, and age are included in supplementary data.<br>For scRNA-seq studies, male patients (age range: 61-76 years) undergoing prostatectomy with either small (n=10) or large (n=4) prostate tissues were included for evaluation of immune cells from the prostate transition zone.
Recruitment	EDW: no recruitment (This was a retrospective chart review)<br>All prostate tissues: Patients are approached prior to a scheduled prostatectomy about participation in the NorthShore Urologic Disease Biorepository. Informed consent is required prior to any use of human tissues.
Ethics oversight	EDW study: NorthShore University HealthSystem institutional review board (IRB).<br>All human prostate tissues: All patient tissues are deidentified, so do not classify as human subjects research. However, patients are enrolled through the IRB-approved NorthShore Urologic Disease Biorepository and Database with informed consent and de-identified clinical annotation. No patients received compensation for participation in these studies.

Note that full information on the approval of the study protocol must also be provided in the manuscript.

Flow Cytometry

Plots

Confirm that:

- [x] The axis labels state the marker and fluorochrome used (e.g. CD4-FITC).
- [x] The axis scales are clearly visible. Include numbers along axes only for bottom left plot of group (a 'group' is an analysis of identical markers).
- [x] All plots are contour plots with outliers or pseudocolor plots.
- [x] A numerical value for number of cells or percentage (with statistics) is provided.

Methodology

Sample preparation	Human prostate transition zone tissues were minced, then digested while shaking at 37°C for 2 hours in 200 U/mL Collagenase I (Gibco) + 1 mg/mL DNase I (Roche) + 1% antibiotic/antimycotic solution in Hank's Balanced Salt Solution. Digestion solution was replaced with TrypLE express dissociation reagent (Gibco) and allowed to shake at 37°C for 5-10 minutes. Digested samples were neutralized in RPMI + 10% FBS, then mechanically disrupted by pipetting repeatedly. Samples were passed through a 100µm cell strainer, then washed. Red blood cells were lysed in a hypotonic buffer, then cells were stained for flow cytometry. Cells were blocked with Human TruStain FcX (Biolegend 422302, lot B270044) prior to antibody staining.
Instrument	BD FACSAria II Cell Sorter
Software	FACS Diva v6.1 (BD) was used for collection and FlowLogic v7 (Miltenyi) was used for analysis.
Cell population abundance	Flow analysis of the cells collected post-sort gave an average of 87% purity in the sort gate across all analyzed events. The vast majority of events that decreased purity were debris in the FSC/SSC plot indicating that any compromised purity was likely from debris rather than contaminating cell types. Single-cell RNA-seq validated minimal contamination with the very small epithelial cell population (<1% total cells).
Gating strategy	CD45+ cell sorting: FSC/SSC gate was set away from the y-axis and outer borders to preliminarily remove debris and doublets. Doublet removal gates were set along the diagonal for FSC-H vs. FSC-A, followed by a rectangular gate in the SSC-W vs. SSC-H plots. Viable cells were gated on the Zombie Violet negative population, and the CD45+ gate was set on the EpCAM-APC vs. CD45-PE plot. Finally, CD45+ cells were gated on CD200-negative and additional EpCAM-negative cells for sorting.<br>Flow analysis of general immune cell populations: FSC/SSC gate was set away from the y-axis and outer borders to preliminarily remove debris and doublets. Doublet removal gates were set along the diagonal for FSC-H vs. FSC-A, followed by a rectangular gate in the SSC-W vs. SSC-H plots. Viable cells were gated on the Zombie Violet negative population, and immune cells gated on CD45-FITC+ gate. CD11b+ cells were indicated as myeloid, and CD11b- cells were gated and further evaluated for other markers. CD11b-CD19+ cells were identified as B cells, while CD11b-CD19- cells were analyzed for T cell markers. CD11b-CD19-CD3+CD4+ were indicated as CD4 T cells, while CD11b-CD19-CD3+CD8+ cells were indicated as CD8 T cells.

- [x] Tick this box to confirm that a figure exemplifying the gating strategy is provided in the Supplementary Information.
